# Diagnostic reasoning in internal medicine: a practical reappraisal

**DOI:** 10.1007/s11739-020-02580-0

**Published:** 2020-12-01

**Authors:** Gino Roberto Corazza, Marco Vincenzo Lenti, Peter David Howdle

**Affiliations:** 1 First Department of Internal Medicine, San Matteo Hospital Foundation, University of Pavia, Pavia, Italy; 2 The School of Medicine, University of Leeds, Leeds, UK; 3 Emeritus Professor of Internal Medicine, Clinica Medica, Fondazione IRCCS Policlinico San Matteo, Piazzale Golgi 19, 27100 Pavia, Italy

**Keywords:** Clinical reasoning, Diagnosis, Internal medicine

## Abstract

The practice of clinical medicine needs to be a very flexible discipline which can adapt promptly to continuously changing surrounding events. Despite the huge advances and progress made in recent decades, clinical reasoning to achieve an accurate diagnosis still seems to be the most appropriate and distinctive feature of clinical medicine. This is particularly evident in internal medicine where diagnostic boundaries are often blurred. Making a diagnosis is a multi-stage process which requires proper data collection, the formulation of an illness script and testing of the diagnostic hypothesis. To make sense of a number of variables, physicians may follow an analytical or an intuitive approach to clinical reasoning, depending on their personal experience and level of professionalism. Intuitive thinking is more typical of experienced physicians, but is not devoid of shortcomings. Particularly, the high risk of biases must be counteracted by de-biasing techniques, which require constant critical thinking. In this review, we discuss critically the current knowledge regarding diagnostic reasoning from an internal medicine perspective.

## Introduction

The burden of disease is always changing [1] and the current COVID-19 pandemic [2] represents an example of this phenomenon. Health systems tend to adapt to the changing burden of disease by developing a variety of strategies including more precise and advanced techniques, such as molecular analysis, genetic mapping, enhanced imaging modalities, or innovative and targeted drugs [3]. However, even in such a time of transition and technological advance, clinical medicine remains an area dominated by uncertainty and probability, and a correct diagnostic reasoning, the pre-requisite for correct management, still remains the cornerstone of good clinical practice.

Making a diagnosis is a cognitive process of logic which involves an element of considering different options (i.e. categorical approximation) and is, therefore, liable to errors that result in adverse patient outcomes [4]. As already mentioned, making mistakes in clinical practice has not been alleviated by progressive technology improvement [5]; on the contrary, an overreliance on new procedures has directly increased the occurrence of such adverse outcomes [6].

A focus on diagnosis is what has been said to define and to differentiate internal medicine from other medical specialties and it has been proposed that the term which may best characterise an internist is “diagnostician” [7]. In internal medicine, patients are more “diagnostically undifferentiated” than in other specialties and, as a consequence, the diagnostic process is susceptible to a higher failure rate [8], largely due to inconsistencies in this reasoning process [9, 10]. Conversely, particularly in internal medicine, characterised by an extremely large body of factual evidence, using a correct diagnostic methodology allows compensation for inevitable case-specific weaknesses [11].

In this paper, we discuss the various stages of the diagnostic process, the prioritisation of different diagnostic reasoning strategies and the procedures by which to detect and prevent the most common errors from a practical, clinical, internal medicine viewpoint.

## The multistage process of diagnosis

**Fig. 1:**
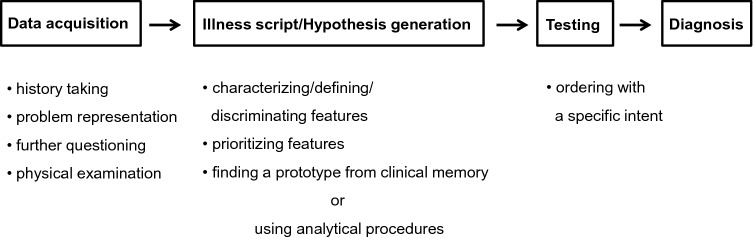
Sequential process of making a diagnosis. Any diagnostic reasoning starts from data acquisition that must be as accurate as possible. Through data acquisition, the physician is able to produce an illness script, generating diagnostic hypotheses, which will be subsequently tested

Clinical diagnosis is a multistage procedure and the sequential phases of this process are shown in Fig. 1. Irrespective of the various clinical settings (including outpatient clinic, hospital ward, or intensive care unit) a diagnosis should be accurate to be maximally effective and efficient in terms of its timeliness and correct use of resources.

Especially in internal medicine, the first and most important phase is the patient-centred interview which takes an holistic and systematic approach. Such an interview aims at collecting structured information and at the same time offers a unique opportunity to gain the patient's confidence, trust, and adherence to guidance. These emotional and empathic aspects of communication, important as they are, are outside the scope of this review and have already been discussed in consensus documents [12].

### Data collection and illness scripts

There are no codified guidelines on how to conduct data collection through a medical interview, rather it should be modelled on the cultural, social and clinical characteristics of each patient. Of course, it is necessary to avoid the collection of an amorphous mix of clinically relevant and irrelevant data and also to avoid focusing on specific organ systems, thus neglecting the patient in his or her entirety. On the other hand, asking the patient what problems led him or her to the visit, when they started, how they evolved over time, what events preceded them, what impact they had on his or her life, represent a proper and shared starting point.

**Fig. 2:**
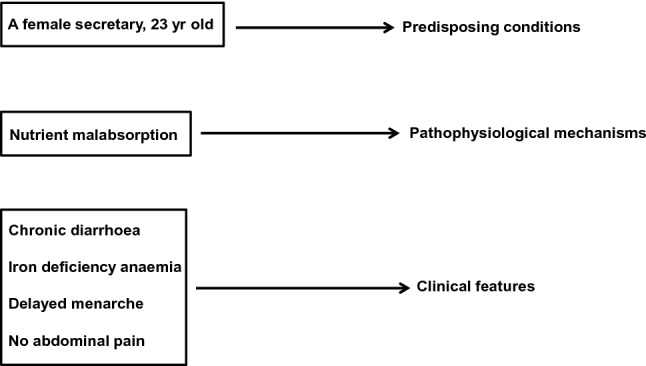
An illness script of a patient with chronic diarrhoea. The illness script is made of three distinct components, namely the predisposing conditions, the pathophysiological mechanisms, and the clinical features. The interaction among the various components generates diagnostic hypotheses

At that point, the patient’s responses are translated into medical equivalents that link the case to formal knowledge [13] and it is on the basis of these collected data that the doctor should seek to outline an early and succinct “problem representation” of the patient [14]. This representation consists in translating his or her present and past history into a meaningful list of clinical problems, detailed by the use of semantic qualifiers, i.e. bipolar descriptors, such as acute/chronic, mild/severe, single/multiple, continuous/recurrent [15]. Each symptom should not be considered as a single element but, where possible, embedded with other related symptoms in a cluster or syndrome [16]. This provisional process should be developed as early as possible, since it must guide further in-depth questions and provide a focus for a physical examination, although that should still be as comprehensive as possible. Thus, through a process of progressively characterising and prioritising the various elements the diagnostician can increasingly define the picture and differentiate it from others, so that an illness script is configured in the clinician’s mind, that is, a story-like narration of disease in which predisposing conditions, pathophysiological mechanisms, and clinical features are articulated [17]. It is a partially automatic experiential process, but precisely for this reason, it is not universally available. The possibility and ease of script triggering depend on the pre-stored expertise of each doctor, that is, on the individual repertoire of knowledge and clinical experience. For example, faced with a young woman with chronic diarrhoea as the main complaint, a defined script, such as that of Fig. 2, will be more easily retrieved by an internist who has already encountered similar cases. Furthermore, illness scripts have a series of inherent limitations. For example, the information contained in a script is not exclusive of it but can belong to several scripts, the activation of one particular script may lead to the activation of a different one due to a sharing effect, and scripts can only interpret the instances of an illness at that particular timepoint and are limited to it [18].

### Models of diagnostic reasoning and hypothesis generation

The configuration in a doctor's mind of a particular script, whether it is correct or not, is essential for the hypothesis generation of disease (Fig. 1), which must subsequently be confirmed or ruled out. These early stages of the diagnostic process are crucial because an appropriate problem representation, to which a consistent and meaningful illness script is connected, avoids random-generated hypotheses based on isolated and clinically irrelevant findings which lead to incorrect conclusions or diagnoses [14].

Although the process of making a diagnosis should theoretically be as flexible and as adaptable as possible and encompass a range of principles and modes of reasoning [19], currently used approaches can be schematically represented into two distinct models, that is, either the intuitive or the analytical approach, the main characteristics of which are listed in Table 1.

**Table 1:** Hallmarks of intuitive and analytical approaches

Intuitive	Analytical
Inductive	Hypothetic-deductive
Unaware	Deliberate
Fast	Slow
Resource-sparing	Resource-intensive

The intuitive approach, inductive and empirically based, makes use, below the threshold of perceptible consciousness, of mental shortcuts (heuristics), allows for quick conclusions, and does not require the use of specific resources. It synthesises the information collected and leads to a consideration of the clinical picture as a whole. Symptoms and findings considered in clusters are quickly and automatically related to a prototype of disease through a pattern recognition process that is already present in the doctor’s mental database [20, 21]. It is clear that for an experienced clinician, the retrieval of encapsulated knowledge, which then leads to a diagnostic hypothesis, starts with data collection and, gradually taking shape, is implemented with the configuration of an illness script.

It must be recognised that this intuitive model rests on a proven scientific background, and has nothing to do with the so-called “gut feeling” which may be used in an initial general assessment by a general practitioner. This consists of an intuitive feeling of alarm (more rarely of reassurance), which usually allows the doctor to distinguish what is urgent from what is not [22]. In the setting of general practice, a very detailed diagnosis is often a less pressing goal than an appropriate and timely referral [23] and, accordingly, the gut feeling has a more prognostic than diagnostic value.

The analytical approach, the other main diagnostic reasoning model, based on a hypothetical-deductive logic, relies on the conscious and deliberate use of evidence-based algorithms or decision trees, which test the relative probability of various hypotheses starting from the signs or symptoms considered as more relevant or typical, rather than from the clinical picture as a whole [24–26]. This second type of approach, which requires more time and use of resources, is instinctively and prudently preferred by novices who have a limited repertoire of clinical experience, or adopted in the case of atypical or rare presentations of disease which are not immediately indicative of a specific illness script. In general, with increasing clinical practice and thereby expertise, the thought processes of younger, less experienced doctors move from the analytical to the intuitive one [27].

We have few data allowing comparison of the diagnostic success between these two different approaches. In a study involving 20 final-year clinical clerks and 20 expert clinicians who were given the same diagnostic questions, the odds of diagnostic success turned out to be greater when strategies of pattern recognition were used, while, as expected, the novices used a hypothetical-deductive way of reasoning [28]. Further studies are, however, necessary before firm conclusions can be reached. It is likely that differences in medical knowledge between the groups have influenced the results more than the adopted strategy and it is not possible to exclude an unconscious sharing of a certain degree of intuitive reasoning in those who had adopted a more analytical approach [20]. On the other hand, it has long been known that, regardless of the general level of clinical expertise, the adoption of one model or the other, often depends on the “content specificity” of the clinical case, since the success in solving a problem is not a strong predictor of being able to solve successfully another of a different nature [29]. In this regard, if we refer to the illness script reported in Fig. 2, while for some doctors it will be necessary to use very costly, time-consuming algorithms that may need invasive procedures for the study of chronic diarrhoea, for others, already content-aware of the problem, the presence of predisposing (gender and age), defining (chronic diarrhoea, iron-deficiency anaemia, history of delayed menarche) or differentiating findings (absence of abdominal pain which minimises the suspicion of irritable bowel syndrome or Crohn’s disease), will quickly and quite obviously promote the hypothesis of celiac disease.

In conclusion, if it is true that we have no evidence about the superiority of the intuitive versus the analytical model, or vice versa, it is equally true that we should not consider these approaches mutually exclusive. For some time, an integrated approach has been proposed, combining the strengths of each [30]. While according to some, such integration would take place in the final stages of diagnostic reasoning with the analytical model prevailing over the intuitive one [31], according to others, the two models constitute a bidirectional continuum of mutual integration, within which the intuitive model is expected to prevail in the early stages of hypothesis generation and the analytical one in the final stages of hypothesis testing [32].

### Assessing post-history probability

Since whichever method of reasoning we use is qualitative in nature, this explains why clinicians are natural Bayesians [33]. Bayes’ theorem allows an estimation of the post-test probability of a certain disease given its pre-test probability (the prevalence of that disease in the underlying population or the clinician's belief about its prevalence) and the accuracy of the test. There is no doubt that only a small minority of clinicians, if any, formally practice in this way [34], and that some of them are unaware of the real prevalence of a disease in relation to individual patient’s characteristics (e.g. family and personal anamnesis, life habits, therapy, symptoms or clinical signs) [35]. Post-test probability of disease depends, in addition to its frequency, on a series of additional data sets (e.g. test results, symptoms, cluster of symptoms) that emerge in the various stages of the diagnostic processing pathway [36].

At any rate, it is by applying such a process to the illness script reported in Fig. 2 that the likelihood of coeliac disease becomes a strong probability.

### Hypothesis testing

This is the third phase of the sequential diagnostic process (Fig. 1). To confirm or not the previously formulated diagnostic hypotheses, and also to quantify the severity of the disease, the physician can be aided by blood tests—from the most common to the most specific functional tests—to assess the extent of organ damage, imaging techniques—from traditional to the most modern—and invasive endoscopic procedures, either separately or in various combinations. The selection of these tests or procedures must not be made fortuitously to hit an unknown target through a “shotgun” approach, as this often leads to results which are difficult to interpret and contradictory to clinical conclusions. Like the previous phases of the diagnostic process, hypothesis testing must be specific and intentional, as well as following the criteria of cost-effectiveness and “choosing wisely” campaigns [37].

## Bias and debiasing

A heuristic process, as we have seen, represents an essential part of intuitive thinking, and is the preferred way of diagnostic reasoning for most expert clinicians. It is a very useful tool, but the clinician must maintain a critical attitude to his or her decision-making to avoid a number of cognitive biases, which can lead to potentially serious consequences for the patient and have been the subject of extensive reviews [38–40]. The most common biases consist of (1) availability bias, i.e. the tendency to consider a diagnosis which is easily retrievable because it is common, exceptional or severe; (2) representative bias, i.e. the tendency to consider only the typical or characteristic manifestations of a disease without taking into account any possible atypical manifestations; (3) confirmatory bias, i.e. the tendency to seek confirmation and to reject disproval to a given hypothesis; (4) anchoring bias, i.e. the tendency to consider correct the diagnosis already formulated in spite of contrary evidence; (5) premature closure bias, i.e. the loss of important information due to the early conclusion of the diagnostic procedure. This latter represents a very frequent bias, although it is usually linked to the analytical approach [41]. Table 2 shows practical examples of the aforementioned biases in relation to the illness script reported in Fig. 2.

**Table 2:** Practical examples of biases in relation to the illness script reported in Fig. 2

Bias	Example
Availability	To consider diarrhoea as a manifestation of irritable bowel syndrome, given its high prevalence in young women, without properly appraising the whole clinical picture
Representative	To consider delayed menarche only as a manifestation of endocrinological disfunction or growth disorders, rather than a possible consequence of longstanding malabsorption
Confirmatory	To disprove the failure to recover anaemia with oral iron supplementation as a possible sign of malabsorption
Anchoring	To consider anaemia as a consequence of metrorrhagia, as previously stated by a gynaecologist
Premature closure	To treat symptomatically diarrhoea and anaemia without performing celiac antibodies and/or intestinal biopsy

As stated above, intuitive thinking often develops below the threshold of awareness; it is not easy, therefore, to avoid such biases. Hence, de-biasing strategies have been developed [39, 40, 42]; however, their initiation in routine clinical practice does presuppose an uncommon critical attitude. Furthermore, most of these de-biasing strategies have been shown to be ineffective in clinical practice. In particular, educational interventions with trainees [43], the use of a de-biasing checklist [44], or slowing down the reasoning process and being more thoughtful [45], did not improve the diagnostic process.

To summarise, being aware of these biases, of the mechanisms that underlie them and of the need to maintain continuous vigilance towards them currently constitutes the safeguard that can contain these errors [46], as well as the integration of the intuitive approach with the analytical one [30]. Other alternatives are not currently available, and what is important is that the possibility of cognitive bias must not, and should not, interfere or limit the use of an intuitive way of diagnostic reasoning.

## Conclusions

It is universally acknowledged that clinical practice is a difficult role and that the bed-side is certainly a more uncomfortable place than the bench-side, given that this latter deals experimentally with one variable whereas the former needs to manage multiple variables at one time within the same patient [47]. In clinical medicine, compared to other natural sciences, levels of certainty are reduced and degrees of freedom are increased and, as a method of analysis, mathematics gives way to statistical probability [48]. The classical characteristic of clinical medicine is represented by the diagnosis, and this is why the fundamentals of diagnostic reasoning should be a core skill taught by medical schools [49].

However, it remains controversial as to whether clinical/diagnostic reasoning should be taught or whether it can only be learned by students at the bedside, independently of teaching staff [50]. Expert teachers are crucial in guiding the identification of conceptual and causal relationships between apparently unrelated findings [51] and in promoting and guiding qualitative feedback on the thought processes of students using metacognition, i.e. the conscious ability critically to monitor one’s thinking [52]. This process of open reflection and feedback is considered the most distinctive feature of “deliberate practice”, and is a leading theory of expertise development and maintenance [53]. It is particularly suited to the practice of internal medicine. However, important though it may be, deliberate practice is not the only predictor of expert performance. Individual ability, such as working memory capacity, i.e. the ways to efficiently store and retrieve knowledge in memory, can be equally important [54].

Internal medicine is the largest medical specialty in the United States [55] and is burdened by a higher rate of diagnostic errors than others [10, 56]. Its main task is to coordinate the care of adult or elderly patients with chronic, complex and often multiple diseases [57]. Because of these characteristics, internal medicine is considered the domain of clinical complexity, to which multiple morbidity, and the patient’s individual characteristics and contextual non-biological factors contribute [58].

Everything that has been discussed above about the various stages of the diagnostic process applies to internal medicine. In particular, aspects related to the need for flexibility and adaptability must be considered including switching from one diagnostic reasoning strategy to another according to contextual needs. What must be stressed is that when faced with complex patients, a reductionist mindset, focused on individual problems, must give way to a way of thinking in terms of the various systems and elements interacting together [59]. This way of reasoning aligns well with the intuitive approach. Considering these systemic aspects of the patient and also their context enables additional predictors to add more detail to the illness script, to facilitate its retrieval, and thus to increase the consistency of the hypothesis.

The internists, therefore, are asked to broaden their critical approach to the patient’s environment and to commit themselves to seeking connections in order to synthesise the diagnostic hypothesis and to rely on their experience however limited it may be. Despite its limitations, that of diagnosis remains an example of science in action [60], in which clinical experience and the maintenance of a critical attitude are certainly important components of this science. We do agree that the experiential component of clinical expertise constitutes a safer guide in the diagnostic process than the knowledge of the latest evidence-based systematic review [13]. Indeed, the clinical competence of the physician cannot be replaced either by the multiplication of technological advances or by evidence-based medicine, of which the numerous limitations are now becoming apparent, but rather all these elements need to be integrated.
